# Herbivore‐induced defenses are not under phylogenetic constraints in the genus *Quercus* (oak): Phylogenetic patterns of growth, defense, and storage

**DOI:** 10.1002/ece3.7409

**Published:** 2021-04-06

**Authors:** Cynthia Perkovich, David Ward

**Affiliations:** ^1^ Department of Biological Sciences Kent State University Kent OH USA

**Keywords:** growth‐differentiation balance hypothesis, phylogenetic comparative methods, phylomorphospace, *Quercus*, resource availability hypothesis, tannins

## Abstract

The evolution of plant defenses is often constrained by phylogeny. Many of the differences between competing plant defense theories hinge upon the differences in the location of meristem damage (apical versus auxiliary) and the amount of tissue removed. We analyzed the growth and defense responses of 12 *Quercus* (oak) species from a well‐resolved molecular phylogeny using phylogenetically independent contrasts. Access to light is paramount for forest‐dwelling tree species, such as many members of the genus *Quercus*. We therefore predicted a greater investment in defense when apical meristem tissue was removed. We also predicted a greater investment in defense when large amounts of tissue were removed and a greater investment in growth when less tissues were removed. We conducted five simulated herbivory treatments including a control with no damage and alterations of the location of meristem damage (apical versus auxiliary shoots) and intensity (25% versus 75% tissue removal). We measured growth, defense, and nutrient re‐allocation traits in response to simulated herbivory. Phylomorphospace models were used to demonstrate the phylogenetic nature of trade‐offs between characteristics of growth, chemical defenses, and nutrient re‐allocation. We found that growth–defense trade‐offs in control treatments were under phylogenetic constraints, but phylogenetic constraints and growth–defense trade‐offs were not common in the simulated herbivory treatments. Growth–defense constraints exist within the *Quercus* genus, although there are adaptations to herbivory that vary among species.

## Graphical abstract

Phylogenetic approaches to understanding trade‐offs between plant defense strategies may help predict patterns of growth, defense, and storage after herbivore damage. We used phylogenetic comparative methods to analyze evolutionary patterns in plant responses in the genus *Quercus*. We found that phylogeny can be used to predict certain response traits, but many responses are species‐specific and patterns do not always exist at the species level.

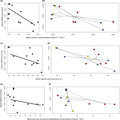

## 1 INTRODUCTION

Herbivory is a key ecological process that acts as a selective pressure and regulates the evolutionary trajectories of plant defenses (Agrawal & Fishbein, 2006; Poelman & Kessler, 2016; Züst et al., 2012). Depending on herbivore pressures, plant defenses may be either constitutive (i.e., fixed) or induced (i.e., activated in response to a stimulus), where these strategies can trade‐off to maximize fitness in a given ecological context (Moreira et al., 2014; Rasmann & Agrawal, 2011). Induced plant defenses are thought to be more costly because energy and resources are diverted away from primary functions to produce defenses, thus creating a growth–defense trade‐off (Campos et al., 2014; Cippolini et al., 2014; Herms & Mattson, 1992; Huang, et al., 2019; Züst & Agrawal, 2017). Growth–defense trade‐offs are also found between growth and *constitutive* defenses (e.g., Züst & Agrawal, 2017). More recent studies have given us a better understanding of the physiological mechanisms that produce a growth–defense trade‐off in plants (Campos et al., 2014; Havko et al., 2016; Züst & Agrawal, 2017; Guo et al., 2018; Ballaré & Austin, 2019). While we are starting to better understand physiological mechanisms and costs that sculpt growth–defense trade‐offs (Guo et al., 2018; Ballaré & Austin, 2019), our understanding of the ecological and evolutionary role of herbivory behind these trade‐offs is less clear (Metcalf, 2016).

### 1.1 Evolutionary theories of plant investment in defenses

Resources are often limited so that plants are unable to attain sufficient nutrients to maximize both growth and secondary physiological processes, such as defense production (Coley et al., 1985; Lorio, 1986; Scogings, 2018). The resource availability hypothesis (RAH) (Coley et al., 1985) and the growth–differentiation balance hypothesis (GDBH) (Herms & Mattson, 1992) are two plant defense theories that can be used together to best predict levels of defenses within an ecological context. The RAH predicts that a plant's ability to access nutrients restricts allocation of those resources so that plants from high‐stress environments will have slower growth rates and will have a greater investment in defenses to minimize herbivory than plants in low‐stress environments (Coley et al., 1985; Grime, 2006; Karban & Baldwin, 1997). Consequently, plants in high‐stress environments are more likely to have evolved higher levels of constitutive chemical defenses than plants in low‐stress environments (Coley, 1988; Grime, 2006). The GDBH hypothesizes that investments in *growth* (cell division and elongation) and *differentiation* (all other metabolic processes, including defense production) are mutually exclusive (Loomis, 1932, 1958). GDBH predicts that plants in low‐stress environments will have a greater investment in growth than defense, whereas plants in high‐stress environments will invest less in growth and more in differentiation (i.e., defense production) (Herms & Mattson, 1992). RAH and GDBH make contradictory predictions about when a plant can provide a maximum defense. RAH predicts that maximum defense will occur when nutrient availability is low (Coley et al., 1985; Grime, 2006). GDBH predicts that a plant's maximum defense will occur at intermediate levels of nutrient availability. That is, when nutrient availability is sufficiently high to synthesize the chemical defenses (Herms & Mattson, 1992), but not high enough that replacement of lost tissues is less costly (Endara & Coley, 2011; Glynn et al., 2007; Hattas et al., 2017; Scogings, 2018).

Due to trade‐offs between growth and defense, plants may differentiate between regrowth and defense strategies to maximize fitness. For example, location of meristem damage may cause differential allocation of resources to growth and defense (Bonser & Aarssen, 1996; Ward, 2010). Plants that express apical dominance (the main central shoot of a plant grows more quickly than auxiliary shoots) may lead to compensatory regrowth of the apical shoot when damaged (Aarssen, 1995; Ballaré & Austin, 2019; Ward, 2010). Contrastingly, auxiliary shoots may be produced when the apical shoot is damaged, leading to an increase in auxiliary shoot growth (Gadd et al., 2001; Ward, 2010). In the genus *Quercus*, which are predominantly forest‐dwelling species, defending the apical meristem is more beneficial than defending the auxiliary meristems to ensure access to light (*sensu* Banta et al., 2010).

### 1.2 Phylogenetic constraints on plant defenses

It is important in comparative ecological studies among species to also consider phylogenetic information to address the statistical non‐independence between species (Ackerly & Donoghue, 1995; Mundry, 2014; Pennell & Harmon, 2013). Species are descended from one another in a hierarchical fashion that violates assumptions of independence of data points (Blomberg et al., 2003; Felsenstein, 1985). More closely related species will have similar defensive chemistry because of shared evolutionary relationships (Craft et al., 2013; Ehrlich & Raven, 1964; Pearse & Hipp, 2009). Previous research has investigated the influence of phylogeny on constitutive (i.e., fixed) and induced defenses (i.e., activated by an herbivore), but it is unknown how phylogeny influences growth–defense trade‐offs of these two modes of plant defense. However, recent studies have suggested that there are few phylogenetic constraints on plant responses to herbivory (Endara et al., 2017; Moreira et al., 2018; Rasmann & Agrawal, 2011) which questions the importance of phylogenetic constraints on patterns of plant defenses. Moreover, none of these previous studies have manipulated the locations and intensities of herbivory (e.g., Pearse & Hipp, 2012; Moreira et al., 2018).

In this study, we assessed the response traits in *Quercus* (oaks). *Quercus* deploy a wide range of potential antiherbivore chemical defenses (e.g., Cavender‐Bares et al., 2004; Feeny, 1976; Hattori et al., 2004; Moctezuma et al., 2014; Pearse & Hipp, 2012). Several studies have also suggested that *Quercus* species alter nitrogen investment and the distribution of non‐structural carbohydrates (NSC) in foliage to deter herbivores (e.g., Forkner & Hunter, 2000; Peschiutta et al., 2018; Rieske & Dillaway, 2008). Nitrogen concentrations have been shown to decrease in leaves when injured (Boo & Pettit, 1975; Frost & Hunter, 2008). *Quercus* species prioritize the storage of NSC relative to growth and reproduction when defoliated (Wiley et al., 2017). This prioritization is due to the essential role of NSC in regrowth and the production of structures such as new leaves and branches (Fornara & Du Toit, 2008). *Quercus* traits often show phylogenetic patterns due to the evolutionary convergence of *Quercus* phenotypic traits (Cavender‐Bares et al., 2004, 2015). We were interested in determining whether growth–defense trade‐offs exist in the *Quercus* genus and how phylogeny influences strategies among species. We used control treatments to simulate constitutive modes of defense and manipulated location and intensity of damage to evaluate induced modes of defenses in 12 *Quercus* species (Felton, 2008; Giordanengo et al., 2010). We sought to (i) assess phylogenetic constraints on constitutive and induced modes of defense, (ii) assess growth–defense trade‐offs under various degrees of herbivory, and (iii) evaluate patterns of responses without phylogenetic considerations. Moreover, we predicted that due to the energetic costs involved in defense production, investment in defense should increase as severity of damage to tissues increases (Kessler, 2015; Neilson et al., 2013). This will lead to the production of an inducible defense rather than a constitutive (fixed) defense. As predicted by GDBH, we expect to find a trade‐off between growth and defense so that as a plant's defense production increases, the plant's growth rate will decrease. Additionally, increased damage should increase allocation of NSC to belowground storage (Wiley et al., 2017), decreasing leaf NSC concentrations. Finally, we hypothesized that more closely related species will demonstrate similar patterns of growth, defense, and nutrient allocation strategies in response to varying location and intensity of simulated herbivory.

## 2 MATERIALS AND METHODS

### 2.1 *Quercus* taxa, phylogeny, and herbivory treatments

**FIGURE 1:**
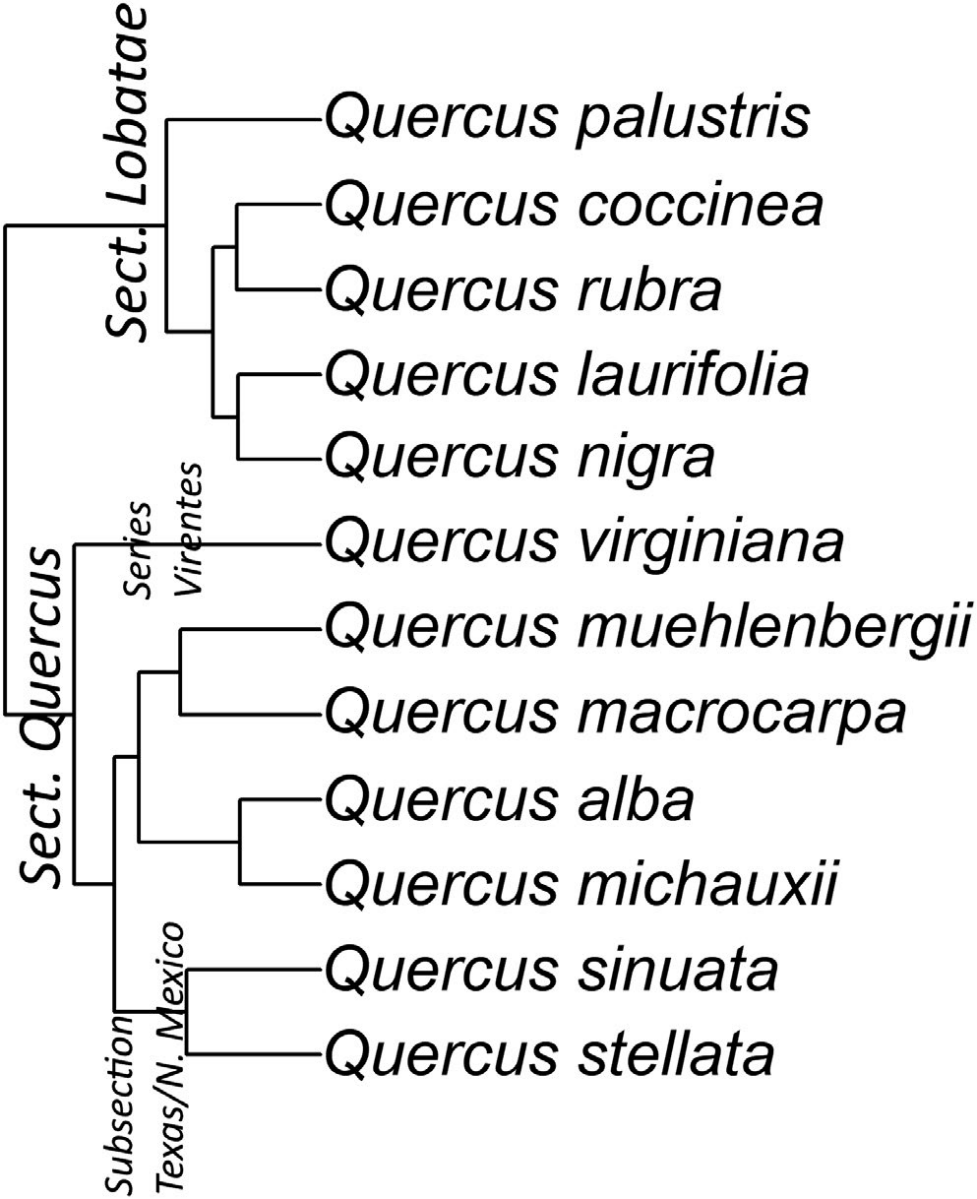
The phylogenetic relationship of 12 oak species used in this study. Phylogenetic information pruned from complete *Quercus* phylogeny by Hipp et al. (2018)

**FIGURE 2:**
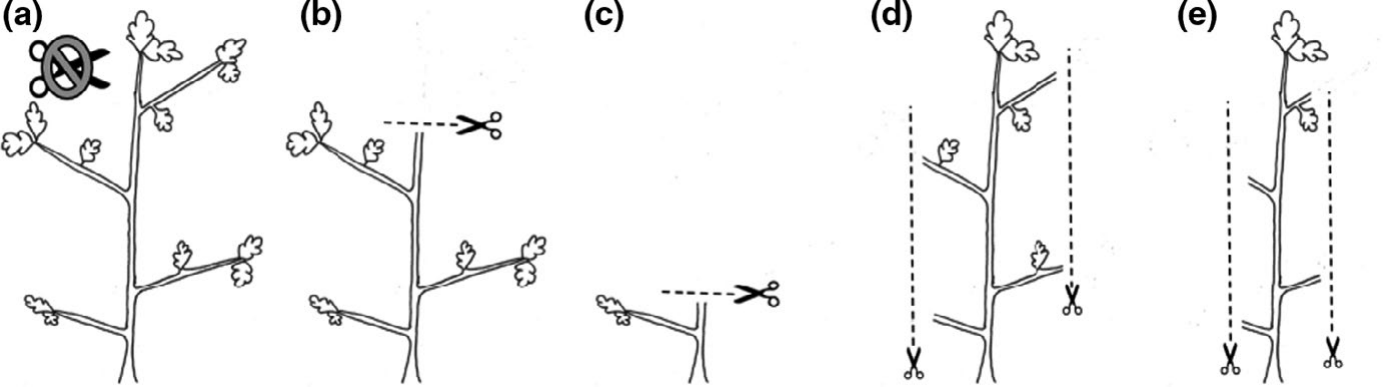
Diagram of the treatments applied to the oak saplings. The treatments include (a) control, (b) 25% apical removal, (c) 75% apical removal, (d) 25% auxiliary removal, and (e) 75% auxiliary removal

Using a well‐resolved phylogeny of the American oak clade (Hipp et al., 2018), we chose 12 species (pruned tree shown in Figure 1) that spanned the phylogeny to obtain a representation of the biogeographical and environmental diversity of the genus *Quercus*. Due to the lack of availability of saplings of certain taxa, we sampled from three of the five major groups in the American oak clade (as defined by Manos et al. (1999) and Hipp et al. (2018)). We sampled *Q*. *coccinea*, *Q*. *laurifolia*, *Q*. *nigra*, *Q*. *palustris*, and *Q*. *rubra* from *Quercus* section *Lobatae*; *Q*. *virginiana* from *Quercus* section *Quercus* series *Virentes;* *Q*. *alba*, *Q*. *macrocarpa*, *Q*. *michauxii*, and *Q*. *muehlenbergii* from *Quercus* section *Quercus*; and *Q*. *sinuata* and *Q*. *stellata* from *Quercus* section *Quercus* subsection Texas/northern Mexico (see Figure 2 Hipp et al., 2018). We followed the nomenclature described by the Oaks Names Database (Trehane, 2007). Interspecific hybridization is common within certain species’ combinations in the genus *Quercus* (Petit et al., 2004; Rushton, 1993), so we avoided species that are known to result from hybridization (e.g., *Q*. *schuettei* was avoided because it is a species that is known to be a hybrid of *Q*. *macrocarpa* and *Q*. *bicolor* (Bray, 1960)). These 12 species of *Quercus* represent a broad spectrum of environmental and ecological diversity within the genus (Hipp et al., 2018).

*Quercus* saplings were purchased from Mossy Oak Nativ Nursery in West Point, MS, United States. We used saplings that were the same age to avoid adaptive responses to damage caused by ontogenetic differences (Gruntman & Novoplansky, 2011). We applied five treatments to mimic variations in location and intensity of simulated herbivory. Each species received all five treatments, which were replicated five times for a total of 25 individuals per species. The five treatments were as follows:

1. Control: no removal of tissues (Figure 2a).
2. 25% apical removal: removal of the dominant apical meristem and 25% apical shoot (Figure 2b).
3. 75% apical removal: removal of the dominant apical meristem and 75% apical shoot (Figure 2c).
4. 25% auxiliary removal: removal of all apical meristems (except for dominant meristem) and 25% of auxiliary shoots (Figure 2d).
5. 75% auxiliary removal: removal of all apical meristems (except for dominant meristem) and 75% of auxiliary shoots (Figure 2e).

### 2.2 Measurements of Quercus defensive traits

Trees were harvested one year after treatment application. For chemical defense traits, leaf and root samples were dried in an oven at 65°C for 48 hr until plant tissues were completely dry. To assess possible differential investments in different types of tannins, we measured total polyphenols and two types of tannins (tannins constitute a type of polyphenol). Polyphenols and tannins were extracted from the oven‐dried plant tissues (Hagerman, 1988) using a 70% acetone solvent (Graca & Barlocher, 2005; Hagerman, 2011). Once extracted, total polyphenol concentrations in the *Quercus* tissue were analyzed using the Prussian blue assay (Price & Butler, 1977) with modifications for use on a microplate reader (Hagerman, 2011). We used gallic acid as a standard (gallic acid equivalents “G.A.E.”). Total tannin concentrations in the *Quercus* tissue were analyzed using the radial diffusion assay and standardized against tannic acid (tannic acid equivalents “T.A.E.”) (Hagerman, 1987). Condensed tannin concentrations were analyzed using the acid butanol assay for proanthocyanidins (Gessner & Steiner, 2005; Hagerman, 2011) and standardized against quebracho tannin (quebracho equivalents “Q.E.”). Note that there are no unique concentrations for polyphenols or tannins, so they are expressed as equivalents of a specific polyphenol or tannin (Hagerman, 2011).

To calculate trichome density, a hole punch was used to punch disks of 7 mm diameter from each leaf. The disks were placed under a microscope lens, and trichome density was calculated as the number of trichomes/dry mass (g) of the 7 mm disk. The average number of trichomes/dry mass (g) of the three disks from each sapling was recorded as the trichome density.

### 2.3 *Quercus* growth and leaf morphology

After treatments were applied, individual *Quercus* tree growth was measured. We measured the height of the apical shoot (*height*) and the lengths of all auxiliary shoots (*auxiliary* *growth*). The *Quercus* saplings were kept in a greenhouse under optimal conditions for one year. Growth measurements for each individual tree were measured biweekly for analysis of relative growth rates. Relative growth rates were calculated for each growth variable (height and auxiliary growth) defined as *RGR* in the equation:

$$RGR=\frac{\ln\left(W_{2}\right)‐\ln\left(W_{1}\right)}{t_{2}‐t_{1}}$$

where *W*_1_ and *W*_2_ are a measurement of the plant's height or auxiliary growth at times *t*_1_ and *t*_2_. RGR calculations minimize bias caused by variance in initial measurements of plant size (Hoffmann & Poorter, 2002; Rees et al., 2010). All growth measurements were taken biweekly throughout the year following treatment application. The final growth measurements were taken once trees were harvested, one year after treatments were applied.

Leaf morphological samples were taken during harvesting, one year after treatment application. Leaves were scanned on a CI‐202 leaf area meter from CID Bio‐Science. After scanning, leaves were dried and weighed. We measured specific leaf area (leaf area divided by the leaf's dry weight), leaf aspect ratio (maximum leaf breadth/maximum leaf length), and leaf shape factor (leaf area/perimeter) by removing three leaves from each sapling and following leaf measurement protocols, as described by Lu et al. (2012).

### 2.4 *Quercus* nutrient allocation responses

The samples were tested for the concentration of total non‐structural carbohydrates using the method by Fournier (2001) that uses a phenol‐sulfuric acid solvent for a colorimetric reaction of sugars and starches extracted from leaf tissues (see Tomlinson et al., 2013). Non‐structural carbohydrate analyses were done in a single laboratory to avoid differences from varying laboratories and techniques (Landhäusser et al., 2018). Nitrogen was analyzed using a rapid N exceed^®^ nitrogen analyzer.

### 2.5 Statistical analysis

#### 2.5.1 Measuring phylogenetic signal and phylogenetic correlations

Phylogenetic comparative methods (PCM) are statistical tools that are commonly used to address the issue of non‐independence among data points (Ackerly, 2009; Felsenstein, 1985; Forthman & Weirauch, 2018; Pennell et al., 2016). Access to phylogenetic information is a major advance in developing PCM that places an emphasis on detecting phylogenetic signal (Mounce et al., 2018; Pennell et al., 2016). Phylogenetic signal ascertains whether there is an effect of molecular phylogeny on any particular trait using phylogenetic distances (Blomberg et al., 2003; Revell et al., 2008).

We calculated phylogenetic signal using a phylogenetic generalized least squares (PGLS) regression (Cornwell & Nakawagaw, 2017; Garland, 1989). We accounted for within‐species variation in the PGLS regressions by using the *pgls*.*Ives* function (Ives et al., 2007) in the *phytools* package (Revell, 2012) in R version 3.6.0 (R Development Core Team, 2019). Following the recommendations of Münkemüller et al. (2012), we report Blomberg's *K* because of its suitability for use with relatively few species (Blomberg et al., 2003). A value of *K* < 1 indicates that species are less similar than expected by phylogenetic relationships and do not follow the Brownian model of evolution; a value of *K* > 1 indicates a greater similarity between species than predicted by the Brownian model. It is important to stress that even a non‐significant value does not necessarily mean that there is no phylogenetic signal, especially in relatively small data sets (Münkemüller et al., 2012). Phylogenetic correlations between response variables were performed within each treatment using independent contrasts (Garland et al., 1999; Pagel, 1999). To account for the possibility of spurious correlations, we performed a Bonferroni correction to adjust the α (Conneely & Boehnke, 2007). This method divides the α by the number of correlations (*n* = 5) to counteract the problem of multiple comparisons. We created *phylomorphospace* plots to project the phylogeny onto the correlation of the two variables being analyzed (Sidlauskas, 2008) to visualize how the data points are phylogenetically related and to visualize how trade‐offs were influenced by phylogeny. We used the *phytools* package (Revell, 2012) in R version 3.6.0 (R Development Core Team, 2019) for these *phylomorphospace* plots.

#### 2.5.2 Statistical analysis ignoring phylogeny

For those variables showing no phylogenetic signal, we first evaluated trait responses across the 12 species using a multivariate analysis of variance (MANOVA) for multiple dependent variables (i.e., height, auxiliary growth, leaf total polyphenol, total tannins, condensed tannin concentrations, and root and leaf non‐structural carbohydrate concentrations) to minimize type I statistical error. Thereafter, we used univariate ANOVA (and Scheffe *post* *hoc* tests) for each significant response variable. The model included herbivory treatment as a fixed factor and species as a random factor. Both MANOVA and ANOVA tests were run using IBM SPSS version 26 software (IBM Corp, 2019). To better understand responses across species, we used a nonparametric sign test (Siegel & Castellan, 1981) to analyze overall trends. Trait variances often display trends that give insights into ecological and evolutionary processes that are not always visible when analyzing mean effects alone (Sánchez‐Tόjar et al., 2020).

## 3 RESULTS

### 3.1 Phylogenetic constraints on constitutive and induced modes of defense

We found a significant phylogenetic signal for constitutive concentrations of total tannin (i.e., in control treatments; Table 1). However, there was no evidence of a significant phylogenetic signal for inducible concentrations of total tannin (i.e., simulated herbivory treatments; Table 1). Trichome production was the only inducible morphological defense that showed a significant phylogenetic signal (Table 1). We found that there was a significant phylogenetic signal for apical relative growth rates (aRGR) in control treatments, but not in herbivory treatments (Table 1). There was also a significant phylogenetic signal for constitutive and induced leaf aspect ratios (Table 1). Similarly, induced specific leaf area and leaf shape showed a significant phylogenetic signal (Table 1).

**TABLE 1:** Results of phylogenetic least squares regression with Blomberg's *K* with significance of phylogenetic signal reported

	Control		25% apical removal		75% apical removal		25% auxiliary removal		75% auxiliary removal	
	*K*	*p*	*K*	*p*	*K*	*p*	*K*	*p*	*K*	*p*
Growth responses										
RGR height	0.836	0.043*	0.639	0.323	0.611	0.387	0.545	0.653	0.538	0.632
RGR auxiliary growth	0.328	0.729	0.843	0.639	0.241	0.272	0.555	0.332	0.537	0.053
Defense responses										
Polyphenols	0.658	0.295	0.751	0.152	0.743	0.214	0.633	0.366	0.528	0.782
Total tannins	1.091	0.013*	0.531	0.686	0.395	0.987	0.686	0.239	0.606	0.412
Condensed tannins	0.455	0.897	0.650	0.305	0.671	0.258	0.685	0.214	0.878	0.038*
Trichome density	0.287	0.316	0.878	0.615	0.456	0.688	0.312	0.999	1.089	0.982
Morphological traits										
Specific leaf area	0.715	0.817	0.841	0.049*	1.067	0.013*	1.003	0.028*	0.886	<0.001*
Leaf aspect ratio	0.963	<0.001*	0.786	0.001*	0.876	<0.001*	1.016	0.039*	0.977	<0.001*
Leaf shape factor	0.313	0.514	0.814	0.018*	1.001	<0.001*	0.971	0.004*	0.999	<0.001*
Nutrient allocation										
Foliar NSC	0.416	0.288	0.770	0.144	0.743	0.187	0.610	0.379	0.502	0.347
Root NSC	0.753	0.165	0.643	0.400	0.520	0.540	0.562	0.085	0.537	0.689
Foliar nitrogen	0.540	0.132	0.643	0.068	0.510	0.740	0.641	0.361	0.646	0.323

Note

Significant values indicated in bold with “*”.

### 3.2 Growth–defense correlations

**FIGURE 3:**
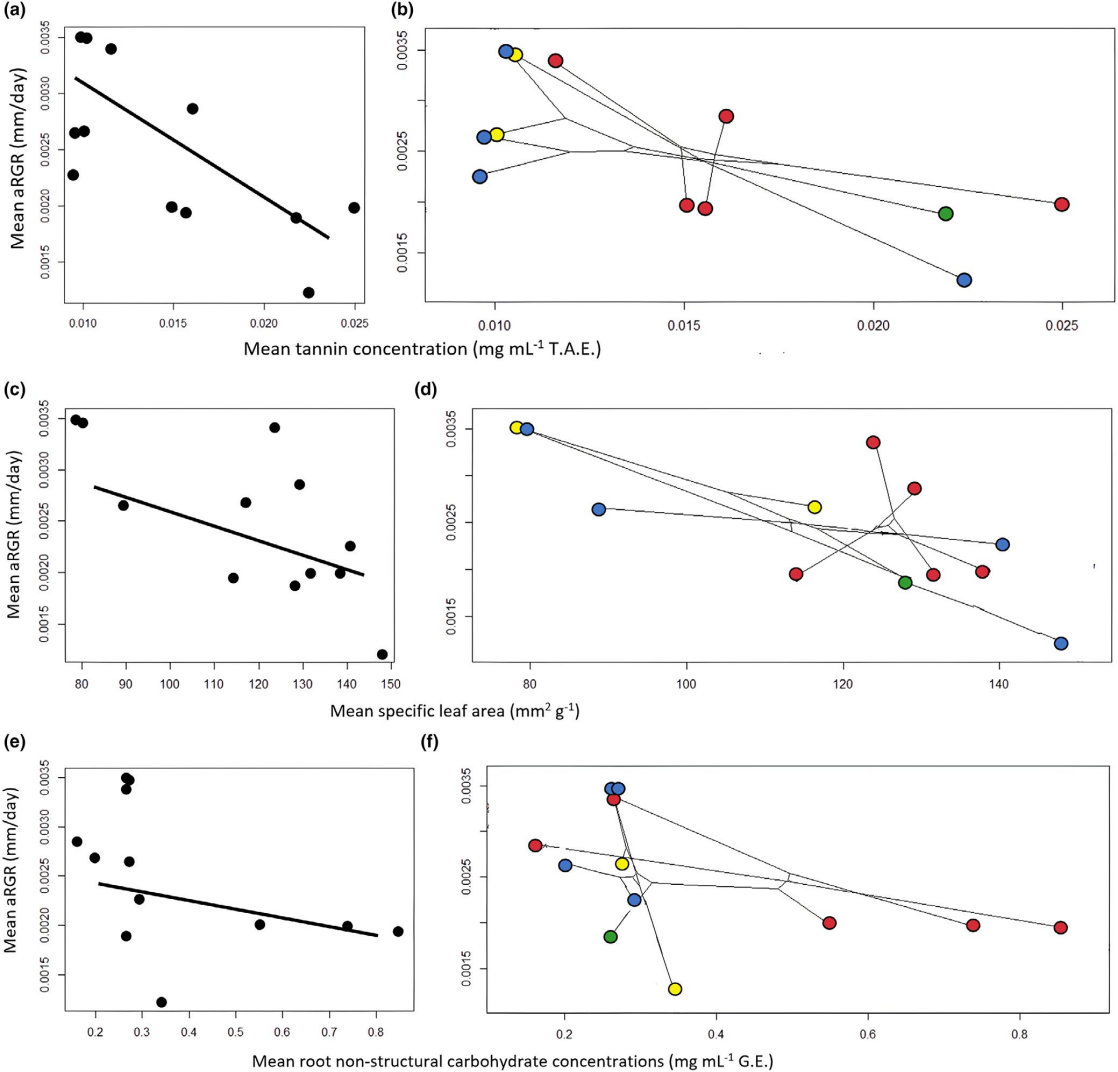
Trade‐offs between *Quercus* constitutive traits and phylomorphospace projections of the *Quercus* *phylogeny*. Each data point represents an individual species’ growth and constitutive trait plotted in morphospace (for more details, see Methods). a) The phylogenetic trade‐off between growth (i.e., apical shoot relative growth rate) and constitutive chemical defenses (tannin concentration) (*r* *=* *‐0.71, p = 0.01)* b) A phylomorphospace plot of (a). c) The trade‐off between growth and leaf morphology (specific leaf area) (*r* *= ‐0.71, p = 0.007* d) A phylomorphospace plot of (c). e) The trade‐off between growth and constitutive nutrient allocation (root non‐structural carbohydrate storage) (*r* *= ‐0.49, p = 0.019)* f) A phylomorphospace plot of (e). Particular colors represent the same section of the phylogeny. Circles in red = *Lobatae*, blue = *Quercus*, green = *Quercus* series *Virentes*, and yellow = *Quercus* subsection Texas/N. Mexico. “aRGR” = apical shoot relative growth rate, “T.A.E.” = tannic acid equivalents, “G.E.” = glucose equivalents.

Using independent phylogenetic contrasts, we found a significant trade‐off (strong negative correlation) between growth and defense (*r* = −0.71, *p* = 0.01; Figure 3a), growth and investment in leaf morphology (*r* = −0.73, *p* = 0.007; Figure 3b), and growth and nutrient allocation (*r* = −0.49, *p* = 0.019; Figure 3c). However, these trade‐offs were not observed in responses induced by simulated herbivory. We also found a significant positive correlation between trichome production and growth (*r* = 0.513, *p* = 0.033). We did not find any other positive correlations between growth and defense responses (*r* range −0.113 to 0.396, *p* range 0.039 to > 0.05).

### 3.3 *Quercus* responses ignoring phylogeny

We assessed *Quercus* responses, ignoring phylogeny, using a MANOVA. After one year of regrowth, *Quercus* species showed significant responses to location of simulated herbivory, intensity of simulated herbivory, and the interaction of location and intensity of simulated herbivory (Table 2). The patterns of responses to location and intensity of simulated herbivory differed significantly among species (Table 2). We used *post* *hoc* univariate ANOVA to further analyze significant results (discussed below).

**TABLE 2:** MANOVA results for the effects of location and intensity on oak growth, defenses, and nutrient allocation

Treatment	Wilks’ λ	*F*	*p*
Location of simulated herbivory (apical versus auxiliary)	0.825	4.661	<0.001*
Intensity of simulated herbivory (25% versus 75%)	0.861	3.566	<0.001*
Location × intensity of simulated herbivory	0.844	4.072	<0.001*
Species (random effect)	0.004	13.942	0.019*

Note

Reported *F* values are equivalent *F* values based on Wilks’ λ. Significant values indicated in bold with “*”.

#### 3.3.1 *Quercus* defense responses

**FIGURE 4:**
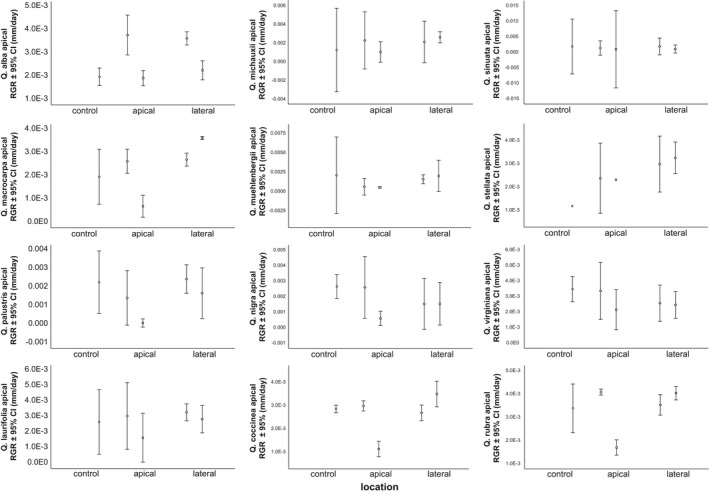
Apical shoot relative growth rate of individual *Quercus* species plotted by location and intensity of simulated herbivory. Scheffé *post hoc* tests result in p values < 0.05 for all species, except *Q. michauxii* (*p* = 0.08), *Q. sinuata* (*p* = 0.16), *Q. palustris* (*p* = 0.38), and *Q. rubra* (*p* = 0.47). 95% C.I. = 95% confidence interval, circle = control, triangle = 25% removal, square = 75% removal.

There was no significant change in induced concentrations of total polyphenols or total tannins (Table 3). In general, albeit not statistically significant, members of *Quercus* section *Lobatae* (*Q*. *coccinea*, *Q*. *laurifolia*, *Q*. *nigra*, *Q*. *palustris*, and *Q*. *rubra*) decreased condensed tannin production when damaged, and members of *Quercus* section *Quercus* (*Q*. *alba*, *Q*. *macrocarpa*, *Q*. *michauxii*, and *Q*. *muehlenbergii*) increased or did not change investments in condensed tannins (Table 3; Figure 4).

**TABLE 3:** ANOVA results of the effects of location and intensity on oak growth, defenses, and nutrient allocation

	Location of Simulated Herbivory (apical versus auxiliary)		Intensity of Simulated Herbivory (25% versus 75%)		Location X Intensity of Simulated Herbivory	
	*F*	*p*	*F*	*p*	*F*	*p*
Growth responses						
RGR height	19.792	<0.001*	21.411	<0.001*	23.909	<0.001*
RGR auxiliary growth	0.001	1.000	0.042	0.838	0.854	0.356
Defense responses						
Polyphenols	2.054	0.153	0.811	0.369	0.885	0.348
Tannins	0.208	0.649	4.170	0.051	0.058	0.810
Condensed tannins	0.933	0.335	6.149	0.014*	<0.001	0.995
Trichome density	0.481	0.489	0.960	0.328	1.398	0.597
Morphological traits						
Specific leaf area	0.406	0.524	0.573	0.450	1.928	0.166
Leaf aspect ratio	0.280	0.607	2.695	0.029*	3.026	0.108
Leaf shape factor	1.105	0.005*	0.407	0.536	0.016	0.900
Nutrient allocation						
Foliar NSC	2.054	0.153	0.811	0.369	0.885	0.348
Root NSC	2.368	<0.001*	2.870	0.092	3.156	0.077
Foliar nitrogen	0.280	0.597	0.115	0.735	1.166	0.281

Note

Significant values indicated in bold with “*.” Growth responses were measured as relative growth rates (RGR), and nutrients include total nonstructural carbohydrates (NSC).

#### 3.3.2 *Quercus* growth and leaf morphology

**FIGURE 5:**
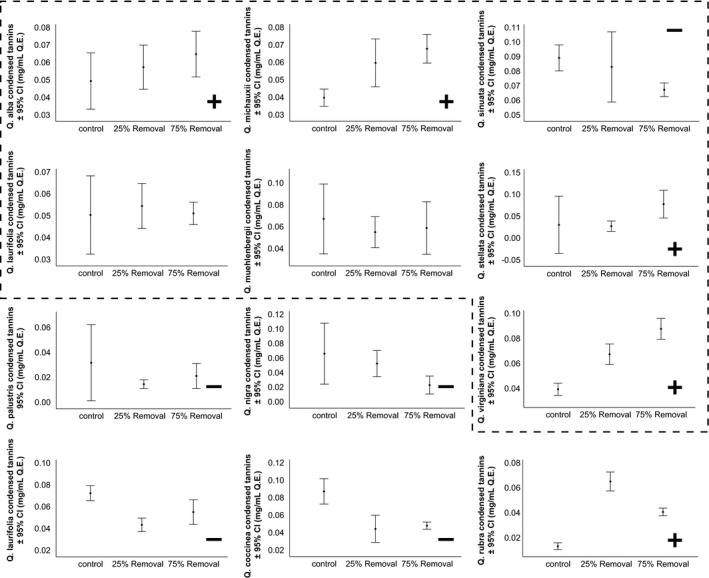
The mean condensed tannin concentrations of individual *Quercus* species in response to location of simulated herbivory (apical vs auxiliary) in each treatment. Taxa inside the black dotted line belong to *Quercus* section *Quercus* (various series) of the *Quercus* phylogeny and those outside the dashed lines belong to the *Lobatae* section. “+” = a positive trend from the control, “‐” = a negative trend from the control. For two species (*Q. macrocarpa* and *Q. muehlenbergii*), there was no significant effect and consequently neither a “+” nor “‐” is indicated. Q.E. = quebracho equivalents (see methods); 95% C.I. = 95% confidence interval.

Regardless of location of simulated herbivory, saplings (except for *Q*. *alba*) with 25% removal of tissue did not increase apical shoot relative growth rates (aRGR) in response to simulated herbivory (Table 3; Figure 5). A nonparametric sign test (Siegel & Castellan, 1981) showed a trend of decreased aRGR when 75% of tissue was removed (sign test: *p* = 0.02; Figure 5) compared to control and 25% removal treatments (Figure 5).

**FIGURE 6:**
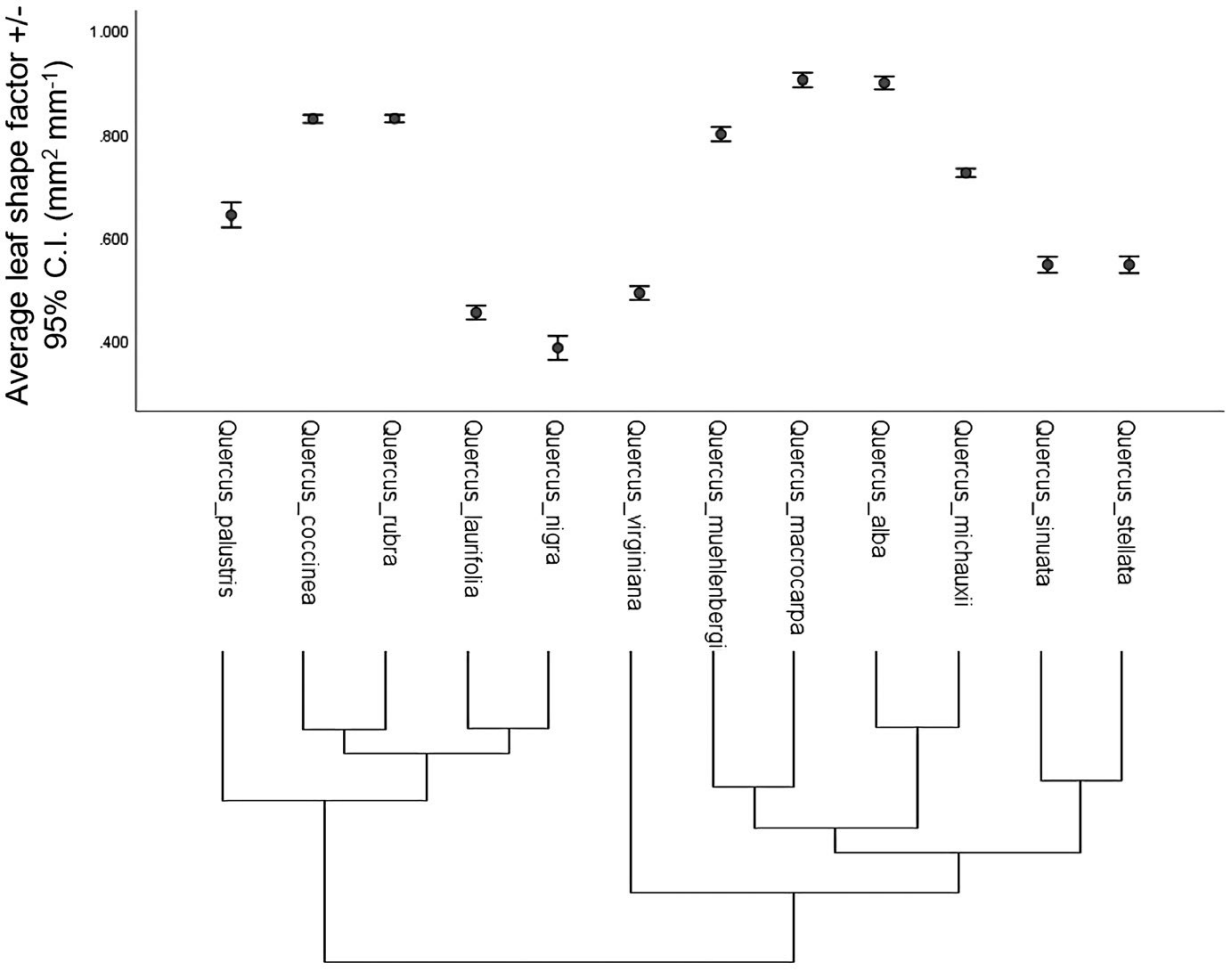
The average leaf shape factor for all treatments was significantly affected by phylogeny for all simulated herbivory treatments. The phylogenetic tree shows that the relationship between species and more closely related species frequently have similar specific leaf areas (mm^2^/g)

*Quercus* specific leaf area did not change in response to simulated herbivory (Table 3). Leaf aspect ratio increased (leaves became more elongated) with 75% removal of tissue, regardless of damage location, in five species (*Q*. *coccinea*, *Q*. *laurifolia*, *Q*. *nigra*, *Q* *stellata*, and *Q*. *virginiana*) (Table 3). Leaf shape factor decreased (i.e., leaves became smaller) in seven *Quercus* species (*Q*. *macrocarpa*, *Q*. *michauxii*, *Q*. *muehlenbergii*, *Q*. *nigra*, *Q*. *sinuata*, *Q*. *stellata*, and *Q*. *virginiana*) when damaged at the apical shoot regardless of the amount of tissue removed (Table 3; Figure 6).

#### 3.3.3 *Quercus* nutrient allocation responses

*Quercus* species had similar constitutive concentrations of NSC in root storage (intraspecific variation ranging from 0.1 to 0.5 mg/ml), with the exception of *Q*. *stellata* (0.9 ± 0.01 mg/ml), which differed significantly from the remaining eleven species (*p* < 0.01). All *Quercus* species, except for *Q*. *stellata*, increased NSC concentrations in root storage when damaged, regardless of the intensity of the simulated herbivory (Table 3). We found no changes to foliar nitrogen concentrations (all *p* values > 0.05; Table 3).

## 4 DISCUSSION

*Quercus* defenses are driven by evolutionary selective pressures (such as herbivory) and the environment (Ackerly, 2002; Firmat et al., 2017; Mitter et al., 1991; Pearse & Hipp, 2012). Having a broad geographic distribution, the genus *Quercus* contends with many environments containing diverse herbivore pressures (Cavender‐Bares et al., 2004, 2016). We found that most of the effects of simulated herbivory were not explained by phylogeny. However, variations in constitutive relative growth rate and total tannin concentrations were explained by phylogenetic relationships. With certain locations and intensities of herbivory, more closely related species shared similar investments in growth and induced leaf morphological traits, but phylogeny did not explain patterns of induced concentrations of total tannins. The results of this study, and those of previous studies (Moreira et al., 2018; Pearse & Hipp, 2012), suggest that constitutive chemical defenses and inducible morphological traits are under significant phylogenetic constraints, but inducible chemical defenses appear to be species‐specific.

*Quercus* chemical defenses, such as tannins (Feeny, 1970; Rossiter et al., 1988; Visakorpi et al., 2019), may often be upregulated as an inducible defense against herbivores (Moctezuma et al., 2014; Rohner & Ward, 1997; Ward, 2006). Furthermore, *Quercus* may alter the types of tannins present, such as condensed and hydrolysable tannins, as chemical defenses against specialist herbivores (Clausen et al., 1992). We found that some *Quercus* species, such as *Q*. *alba* and *Q*. *michauxii,* increased condensed tannin production relative to the amount of tissue removed (Figure 5). We note that many studies have found defenses are induced by specific salivary enzymes and proteins (Berman, 2002; Rooke, 2003; Ward et al., 2020). However, herbivore saliva does not always induce these defenses (Keefover‐Ring et al., 2015), and defenses are often induced without such catalysts (Huang et al., 2019).

### 4.1 How much variance in phenotypic traits does phylogeny explain?

In this study, we asked whether phylogenetic constraints could explain patterns of growth–defense trade‐offs between and within *Quercus* species. Phylogeny and adaptation define two ends of a continuum of biological explanations (Agrawal, 2020; Cavender‐Bares et al., 2016; Leimar et al., 2019; Stearns, 1992). We note that it is crucial to keep the idea of a continuum in mind when interpreting phylogenetic analyses of genera, especially *Quercus*, that have broad, overlapping geographic distributions (McVay et al., 2017; Moreira et al., 2018). Furthermore, a phylogenetic pattern of phenotypic traits does not necessarily indicate that the trait is not adaptive (Agrawal, 2020; Heslop‐Harrison, 2017; Stearns, 1992). Ackerly (2002) explained how ecological sorting processes and selection can lead to adaptive evolution. He further provides a framework for how species’ distributions can lead to patterns of phylogenetic niche conservatism. In this study, we found correlations between phylogenetic relatedness and similarity of life‐history traits in the genus *Quercus* (demonstrated by phylogenetic patterns in leaf morphological traits), similar to studies such as Cavender‐Bares et al. (2004).

Physiology and genetics are two well‐studied sources of constraints on adaptations of plant defenses (e.g., Ballaré & Austin, 2019; Endara et al., 2017; Keith, 2017; Ochoa‐Lopez et al., 2018). Studies focusing on genetic constraints of adaptation often fail to consider limitation and assimilation capacity of resources (Ballaré & Austin, 2019; Mole, 1994), just as studies of physiological constraints often fail to evaluate heritability of traits (Ehrlich et al., 2020; Ward et al., 2012). Both types of constraints further fail to explain differentiation of traits expressed across levels of biological organization due to selective pressures (Barthelemy & Caraglio, 2007; Hahn & Moran, 2016; Züst & Agrawal, 2017). For example, certain plant defenses have been shown to trade‐off with plant growth or reproduction within individual species, but general patterns of plant defense trade‐offs are less frequently recorded across related species (Agrawal & Fishbein, 2006; Peiman & Robinson, 2017; Züst & Agrawal, 2017; Züst et al., 2015). More recent advances in phylogenetics have created a better understanding of patterns of plant defense trade‐offs across biological scales and sparked an interest in phylogenetic constraints of plant defense adaptations (Hinman et al., 2019; Moreira et al., 2018; Pausas & Verdu, 2010). Phylogenetic constraints may not always be present but analyzing ecological variation in a phylogenetic context provides important information, even if phylogenetic signal is not detected (Garland et al., 2005; Losos, 2008).

Plant phylogeny often explains much of the variance in key morphological traits as defense expression (Pearse & Hipp, 2009). Many *Quercus* species undergo leaf morphological changes that may act as defenses against herbivores (Dawra et al., 1988; Moctezuma et al., 2014), but it is often difficult to directly link leaf morphology to defense (Moctezuma et al., 2014). For example, *Q*. *virginiana* has a thick, waxy cuticle that acts as a defense (Eigenbrode & Espelie, 1995), perhaps because insects with smaller mandibles find it difficult to try to cut through the tough cuticle (Raupp, 1985). We found several changes to leaf morphology that were induced by simulated herbivory treatments, making it possible for us to conclude that these traits are related to defense. Furthermore, there is considerable evidence that similar leaf morphologies are a result of phylogenetic relatedness (Hickey & Wolfe, 1975; Kadereit et al., 2006; Oyston et al., 2016). If similar herbivore pressures affect certain lineages more consistently than others, we would expect lineage‐specific adaptations that will reflect a phylogenetic pattern (Donoghue, 1989; Lauder, 1981; Walden et al., 2019). However, several studies (e.g., Moreira et al., 2018; Pearse & Hipp, 2009, 2012), including ours, suggest that the tendencies of species to retain ancestral traits cannot entirely account for variations in inducible chemical traits. Even so, we did find examples of inducible defenses that demonstrate phylogenetic effects. One example is the similarity in condensed tannin production of *Q*. *virginiana*, a species from *Quercus* series *Virentes*, and the closely related *Quercus* section *Quercus* (*Q*. *alba* and *Q*. *michauxii*) (Figure 6). We also found that phylogeny explains some variation of inducible leaf morphological traits. Nonetheless, we also found that condensed tannin production in this genus is also differentiated based on the simulated herbivore pressures. For example, except for *Q*. *rubra*, members of *Quercus* section *Lobatae* (*Q*. *coccinea*, *Q*. *laurifolia*, *Q*. *palustris*, and *Q*. *nigra*) decreased condensed tannin concentrations when 75% of tissues were removed.

### 4.2 What do positive and negative (trade‐off) correlations tell us?

Plant scientists have long considered a cost‐benefit paradigm when trying to better understand plant defenses (Cippolini et al., 2014; Huang et al., 2019; Steppuhn & Baldwin, 2008). In this regard, we would expect trade‐offs to be common. The growth–differentiation balance hypothesis (GDBH), as well as the resource availability hypothesis (RAH; Coley et al., 1985), predicts that slow‐growing plants will have more resources available for investment in defenses because they need to limit loss (Hattas et al., 2017; Herms & Mattson, 1992; Scogings, 2018). This may result in species‐specific trade‐offs and are not generally extrapolatable at the generic level (Agrawal, 2020; Futuyma & Moreno, 1988). Species‐specific trade‐offs may explain some of the patterns we observed in this study. For example, the trade‐off we found between apical shoot relative growth rate (aRGR) and total tannin concentration indicates that even though plants received the same resources, species with slower growth rates invested more in defenses than those with higher growth rates (see Results). Individual species in control treatments in *Quercus* section *Lobatae* tend to invest more in total tannin production and less in aRGR relative to species in *Quercus* section *Quercus* (Figure 3b). However, we did not find the expected trade‐off between aRGR and induced total tannin concentrations in herbivory treatments. The absence of this trade‐off may be due to specific genotype‐by‐environment interactions (i.e., adaptive phenotypic plasticity *sensu* Via et al., 1995; van Kleunen & Fischer, 2004; Ward et al., 2012) within certain species of *Quercus*. Other studies have shown substantial evidence of local adaptation as well as adaptive differentiation of *Quercus* species that are closely related (e.g., Cavender‐Bares & Ramirez‐Valiente, 2017; Gonzalez‐Rodriguez & Oyama, 2005; Valladares et al., 2002). Furthermore, foliar NSC concentrations did not significantly change in any of the *Quercus* species, yet several species (*Q*. *alba*, *Q*. *macrocarpa*, and *Q*. *rubra*) increased aRGR. We postulate that increasing aRGR increases photosynthetic capacity by increasing height and access to sunlight, regardless of the increased re‐allocation to belowground (root) storage in the herbivory treatments. For example, *Q*. *rubra* has been shown to increase photosynthetic rates by up to 22% (Woolery & Jacobs, 2011) and increase NSC concentrations in foliar tissues (Frost & Hunter, 2008) following simulated herbivory. Wiley et al. (2017) also showed that *Quercus* species prioritized NSC root re‐allocation relative to growth in *Q*. *rubra*. Rieske and Dillaway (2008) found that defoliation of *Q*. *velutina* had no effect on relative height or non‐structural carbohydrate reserves, but *Q*. *alba* decreased investments in both relative height and NSC in root reserves.

#### 4.2.1 The role of nutrient acquisition in hiding trade‐offs

Trade‐offs may result from genetic associations between growth/reproduction and defense (antagonistic pleiotropy—reviewed in Hedrick, 1999; Johnson et al., 2015; Keith & Mitchell‐Olds, 2019; Rose, 1982; Wright, 1968) or from optimization strategies regarding nutrient acquisition and allocation (Metcalf, 2016; van Noordwijk & de Jong, 1986). Although antagonistic pleiotropy may be a plausible explanation for trade‐offs in *Quercus* species, an equally plausible hypothesis pertains to differences in nutrient acquisition and allocations to growth and defense (Bochdanovits & de Jong, 2004; van Noordwijk & de Jong, 1986; Ward & Young, 2002). Following the latter hypothesis, a trade‐off will occur if there is a relatively small difference in nutrient acquisition between individual plants and a relatively large difference in the allocation of those nutrients (to growth or defense) between individuals (van Noordwijk & de Jong, 1986). However, if there is a relatively large difference in nutrient acquisition between individual plants and a relatively small difference in nutrient allocation (to growth or defense) between individual plants, a trade‐off will not occur (van Noordwijk & de Jong, 1986). In our study, all individuals received the same nutrients and water, although we removed different amounts of photosynthetic material, resulting in reduced acquisition with greater simulated herbivory. The absence of some trade‐offs may result from the plasticity of traits in species that differ in their acquisition and allocation of resources (Metcalf, 2016; van Noordwijk & de Jong, 1986). Indeed, Armbruster et al. (2004) suggested that intraspecific correlations (positive and negative) between growth and defense traits are indicative of adaptations independent of phylogenetic constraints.

Under the GDBH, we would not expect to find positive correlations between growth and defense traits (Ward & Young, 2002). However, resource acquisition may allow for some individuals to allocate more resources to multiple functions (growth and defense) (van Noordwijk & deJong, 1986; Ward & Young, 2002; Zera & Harshman, 2001). We found a positive correlation between growth rate and trichome density in herbivory treatments, after we controlled for phylogeny. Trichomes have been linked to plant defenses and are energetically expensive to produce (Holeski et al., 2010; Levin, 1973; Tian et al., 2012). Given access to the same resources (water and sunlight), we would expect to see a trade‐off between aRGR and trichome density because of the high cost of producing trichomes (Hare et al., 2003; Levin, 1973; Züst et al., 2011). We speculate that the positive correlation between growth rate and trichome density may be a result of a cascade of responses to increased size, resulting in a positive correlation. Furthermore, increases in trichome density can increase water moisture retention, thus increasing photosynthetic capacity (Brewer & Smith, 1994) and resources available to increase growth rate. More research is needed to understand the nature of the genetic correlations (MacTavish & Anderson, 2020) of *Quercus* traits to determine if positive correlations could infer adaptation of defensive traits, or whether they are simply the consequence of allometric scaling (Falster et al., 2015).

### 4.3 Are leaf traits indicative of induced resistance to herbivory?

Energetic costs associated with the production of constitutive and inducible defenses may be offset by the optimization of multiple metabolic pathways resulting in a trade‐off between types of defenses (Gershenzon, 1994; Neilson et al., 2013). In a meta‐analysis of trade‐offs between various plant defenses, Koricheva et al. (2004) suggested that ecological costs of defense production may cause a differential investment between constitutive and inducible plant defenses. Differential investment in constitutive and induced chemical defenses makes it essential to consider the two types of defenses independently (Martinez‐Swatson et al., 2020). Furthermore, phylogeny often constrains phenotypic expression of constitutive defenses (Moreira et al., 2018; Ralph et al., 2007). Inducible defenses may be under greater species‐specific selective pressures and are more likely to be adaptive (Baldwin, 1999; Koricheva et al., 2004; Moreira et al., 2018). Understanding the costs of defense production in both constitutive and inducible defenses is essential to understanding the evolution of plant defenses (Galman et al., 2019a; Galman et al., 2019b; Martinez‐Swatson et al., 2020). We found that herbivore damage induced a greater aRGR (apical relative growth rate), greater production of condensed tannins and alterations in leaf aspect and leaf ratio, as well as induced non‐structural carbohydrate re‐allocation to root storage. Contrastingly, in our study, we found that polyphenol concentration, total tannin concentration, and trichome density remained constant regardless of damage (i.e., constitutive). Leaf traits that evolved for primary functions, such as specific leaf area (Knight et al., 2006), probably contribute to defense against herbivores as well (Agrawal, 2004). We simulated herbivory allowing us to make assumptions about which morphological traits may be directly related to herbivore defense. Our study suggests that certain induced leaf morphological traits (e.g., specific leaf area, leaf aspect ratio, leaf shape factor) may also be under phylogenetic constraints (see also Pearse & Hipp, 2012). For example, we found that leaf shape factor decreased depending on the location of damage, with closely related species behaving more similarly (Table 3).

## 5 CONCLUSIONS

Our study suggests that a combination of RAH and GDBH predictions construct a better representation of how ecological selective pressures, such as herbivory, affect a plant's investment in growth and defense production (Endara & Coley, 2011; Glynn et al., 2007; Hattas et al., 2017; Martinez‐Swatson et al., 2020; Scogings, 2018). At low levels of resources, plants may be able to do little other than grow, while at intermediate levels of resources, there are sufficient nutrients to grow rapidly and produce chemical defenses (GDBH only) as evidenced by the lack of growth–defense trade‐offs in herbivory treatments. At higher levels of resources, plants may focus on growth‐based strategies and regrow any lost material (Coley et al., 1985; Maron et al., 2014; Pearson et al., 2017). Resource allocation, in addition to herbivore pressures, are likely to be factors that drive adaptations of chemical defense in *Quercus*. Overall, our results show uniquely that there are phylogenetic constraints on growth, constitutive tannin concentrations, and the trade‐off between these two variables. Herbivore‐induced condensed tannin concentrations and leaf morphological traits are also under significant phylogenetic constraints (*K* > 1). However, inducible chemical traits (except for condensed tannin concentrations) are influenced by adaptive selection pressures (*K* < 1). Considering the contrasting findings of previous studies about *Quercus* storage of non‐structural carbohydrates (e.g., Rieske & Dillaway, 2008; Wiley et al., 2017), our study shows that NSC re‐allocation strategies within the genus *Quercus* were related to location of meristem damage and not the intensity. We predicted greater responses to apical shoot damage but found that auxiliary shoot damage consistently caused a greater re‐allocation of NSC storage in the roots. However, the overall relationships between growth and NSC re‐allocation appear to represent species‐specific adaptations to selective pressures imposed by herbivory.

## CONFLICT OF INTEREST

There were no conflicts of interest.

## AUTHOR CONTRIBUTIONS

**Cynthia Perkovich:** Conceptualization (equal); Data curation (equal); Formal analysis (equal); Investigation (equal); Writing‐original draft (lead); Writing‐review & editing (lead). **David Ward:** Conceptualization (equal); Formal analysis (supporting); Funding acquisition (lead); Supervision (lead); Writing‐review & editing (supporting).

## 6 ETHICS STATEMENT

This research was conducted in compliance of all ethical standards of research.

## Data Availability

Data used in this research are published in DRYAD. https://doi.org/10.5061/dryad.fttdz08s1.
